# Computer-Aided Diagnosis in Spontaneous Abortion: A Histopathology Dataset and Benchmark for Products of Conception

**DOI:** 10.3390/diagnostics14242877

**Published:** 2024-12-21

**Authors:** Tahir Mahmood, Zeeshan Ullah, Atif Latif, Binish Arif Sultan, Muhammad Zubair, Zahid Ullah, AbuZar Ansari, Talat Zehra, Shahzad Ahmed, Naqqash Dilshad

**Affiliations:** 1 Division of Electronics and Electrical Engineering, Dongguk University, Seoul 04620, Republic of Korea; tahirmahmood@dongguk.edu; 2 Department of Neurophysiology, Cork University Hospital, T12 DC4A Cork, Ireland; doctarzee@gmail.com; 3 Department of Computer Science and Artificial Intelligence, Dongguk University, Seoul 04620, Republic of Korea; atiflatif3452@gmail.com; 4 Department of Pathology, Jinnah Sindh Medical University, Karachi 75510, Pakistan; binish28@gmail.com (B.A.S.); talatzeh@gmail.com (T.Z.); 5 Interdisciplinary Research Center for Finance and Digital Economy, King Fahd University of Petroleum and Minerals, Dhahran 31261, Saudi Arabia; engr.zubairfarooqi@gmail.com; 6 Division of AI Software Convergence, Dongguk University, Seoul 04620, Republic of Korea; zeeuom@gmail.com; 7 Department of Obstetrics and Gynecology, Ewha Woman’s University, Seoul 07985, Republic of Korea; abu.kim.0313@gmail.com; 8 Department of Electronic Engineering, Hanyang University, Seoul 04763, Republic of Korea; 9 Department of Computer Science and Engineering, Sejong University, Seoul 05006, Republic of Korea

**Keywords:** artificial intelligence, spontaneous abortion, tissue phenotyping, deep learning

## Abstract

Spontaneous abortion, commonly known as miscarriage, is a significant concern during early pregnancy. Histopathological examination of tissue samples is a widely used method to diagnose and classify tissue phenotypes found in products of conception (POC) after spontaneous abortion. **Background:** Histopathological examination is subjective and dependent on the skill and experience of the examiner. In recent years, artificial intelligence (AI)-based techniques have emerged as a promising tool in medical imaging, offering the potential to revolutionize tissue phenotyping and improve the accuracy and reliability of the histopathological examination process. The goal of this study was to investigate the use of AI techniques for the detection of various tissue phenotypes in POC after spontaneous abortion and evaluate the accuracy and reliability of these techniques compared to traditional manual methods. **Methods:** We present a novel publicly available dataset named HistoPoC, which is believed to be the first of its kind, focusing on spontaneous abortion (miscarriage) in early pregnancy. A diverse dataset of 5666 annotated images was prepared from previously diagnosed cases of POC from Atia General Hospital, Karachi, Pakistan, for this purpose. The digital images were prepared at 10× through a camera-connected microscope by a consultant histopathologist. **Results:** The dataset’s effectiveness was validated using several deep learning-based models, demonstrating its applicability and supporting its use in intelligent diagnostic systems. **Conclusions:** The insights gained from this study could illuminate the causes of spontaneous abortion and guide the development of novel treatments. Additionally, this study could contribute to advancements in the field of tissue phenotyping and the wider application of deep learning techniques in medical diagnostics and treatment.

## 1. Introduction

Spontaneous abortion (SA), also known as spontaneous miscarriage, is a common issue encountered during the early stages of pregnancy. It is defined as an involuntary loss of pregnancy before 20 to 24 weeks of gestation [1]. While the exact worldwide incidence of spontaneous abortion is unknown, some studies suggest that it occurs in approximately 10% to 20% of all first-trimester pregnancies, with 80% of these occurring within the first 12 weeks [2]. Furthermore, the risk of SA occurrence directly increases with age, from 10–25% up to 60–70% in pregnant females under 25 years old and over 40 years of age, respectively [3]. Histopathological examination [4] is a well-established and widely used method for the diagnosis and classification of various tissue phenotypes, including those found in products of conception (POC) after spontaneous abortion. This examination involves the examination of tissue samples under a microscope to identify any structural or cellular changes that may provide insight into the underlying causes of spontaneous abortion. The study of tissue phenotypes in POC after spontaneous abortion has the potential to provide important insights into the underlying causes of spontaneous abortion and to inform the development of new treatments and therapies [5].

The analysis of histology in specimens obtained from patients with sporadic or recurrent early pregnancy loss is a standard practice in the management of such patients and has been emphasized in the literature. Examining these specimens and tissue phenotyping plays a crucial role in several ways. For example, it confirms the presence of an intrauterine pregnancy by detecting chorionic villi. It helps in the detection of any previously unknown diseases affecting either the mother or the embryo that require immediate medical attention, such as unusual infections, trophoblastic diseases, or changes indicating the presence of aneuploidy or metabolic storage disease. It also helps in the identification of conditions that may recur in future pregnancies or explain adverse outcomes, such as chronic histiocytic intervillositis, maternal malperfusion, or hereditary settings. Lastly, it provides guidance for the management of future pregnancies, for instance, in cases of hydatidiform mole or extravillous trophoblastic lesions. In addition, the histopathologic examination of specimens from spontaneous or recurrent abortions, whether surgically or medically induced, helps protect obstetricians and gynecologists from medico-legal issues [6,7].

The manual analysis of tissue samples from POC after spontaneous abortion can be a time-consuming and subjective process. In recent years, artificial intelligence (AI)-based techniques have emerged as a promising tool in the field of medical imaging, offering the potential to revolutionize the field of tissue phenotyping and improve the accuracy and reliability of the histopathological examination process [8]. AI-based techniques are designed to learn from large amounts of data and can accurately identify and classify various tissue phenotypes in a fast and efficient manner. By incorporating these techniques into the examination process, there is the potential to improve the accuracy and reliability of the diagnosis and classification of tissue phenotypes in POC after spontaneous abortion. The goal of our work is to investigate the use of AI techniques for the detection of various tissue phenotypes in products of conception after spontaneous abortion and evaluate the accuracy and reliability of these techniques compared to traditional manual methods. The results of this study provide valuable insights into the underlying causes of spontaneous abortion and have the potential to inform the development of new treatments and therapies for this condition. Additionally, the results of this study contribute to the advancement of the field of tissue phenotyping and the wider application of deep learning techniques in medical diagnostics and treatment. This study presents several key contributions to the field of histopathological examination and tissue phenotyping in POC after spontaneous abortion:

- We curated a unique publicly available dataset, HistoPoC, comprising 5666 annotated histopathology images collected from 120 patients at Atia Hospital, Karachi, Pakistan. HistoPoC is believed to be the first of its kind that focuses on spontaneous abortion in early pregnancy, providing a valuable resource for researchers and clinicians working in this domain. This publicly available dataset offers a competitive environment and a benchmarking platform to the related research community;
- This research investigates the application of advanced AI techniques for the detection and classification of various tissue phenotypes in products of conception. We aimed to improve the accuracy and reliability of histopathological assessments compared to traditional manual methods, addressing the subjectivity and inter-observer variability inherent in human examinations;
- For the benefit of the research community, we have made the code, trained models, and dataset publicly available. This facilitates reproducibility, encourages further research, and supports the development of additional AI-based diagnostic tools in medical imaging.

The rest of the manuscript is organized as follows: Section 2 presents the related works, Section 3 describes the materials and methods, Section 4 shows the results, and Section 5 discusses the overall work. The conclusion is presented in Section 6 of the paper.

## 2. Related Work

The study of SA, particularly histopathological examination, has garnered significant attention in recent years. Various research efforts have focused on understanding the incidence, risk factors, and underlying causes of SA through the analysis of tissue phenotypes found in POC. Histopathological methods remain a cornerstone in identifying structural or cellular anomalies that may provide diagnostic insight into pregnancy loss. However, the manual analysis of these tissue samples can be time-consuming, subject to human error, and dependent on the expertise of the examiner. Consequently, recent advancements have aimed at improving the accuracy and efficiency of diagnostic processes through the application of automated techniques and deep learning models. For example, Bukhari et al. [9] investigated the utility of artificial intelligence in the histological diagnosis of POC by focusing on the identification of chorionic villi in tissue specimens. A total of 400 anonymized digital images were analyzed and divided into two groups: 200 images containing chorionic villi and 200 images of decidual tissue. Although the study demonstrated promising results in applying deep learning algorithms for diagnostic accuracy, the main limitation of their work is that the dataset is not publicly available, hindering broader validation and reproducibility of the findings.

Palee et al. [10] proposed a computational image analysis method to differentiate between complete and partial hydatidiform moles, which is essential for effective prognosis and management due to their links to malignant tumors. Their approach combines fuzzy C-means clustering with hue, saturation, and value color space to classify villi into trophoblast and stroma regions. However, the study highlighted that no single morphological criterion is sufficient due to variations in villi size, shape, and proliferation, necessitating a combination of criteria for accurate classification. A key weakness is that this reliance on multiple criteria may complicate diagnosis and limit practical applicability, while inherent variability in histological samples can hinder consistent results across cases. Kohut et al. [11] investigated immunologic causes of miscarriage, hypothesizing that women miscarrying their first pregnancy exhibit a more frequent maternal immune response in placental tissue compared to those with prior full-term deliveries. Researchers reviewed charts of 273 women, selecting 32 who achieved full-term pregnancy after their index miscarriage. The findings revealed that lesions of chronic inflammation and uteroplacental vasculopathy were significantly more common in women with first pregnancy losses (37.5%) compared to those with previous successful pregnancies (0%, *p* = 0.02). A key weakness of the study is its small sample size of 32 patients, which may limit the generalizability of the results. Moreover, reliance on historical medical data could introduce biases in reported outcomes.

Ushakov et al. [12] presented EndoNet, a model that automates H-score. H-score is a semi-quantitative method for assessing protein presence in tissue samples, but it can be time-consuming and limited in accuracy. EndoNet enhances histology slide analysis, improving efficiency and adaptability for pathologists. The study’s limitations include a small dataset that may not represent the full variability of histological samples, potentially impacting generalizability. Additionally, the model’s reliance on annotation quality and lack of transparency may hinder trust and applicability in clinical settings. We searched for public datasets related to this topic, but to the best of our knowledge, none are available. Given this absence, our study will provide a valuable starting point for researchers interested in exploring the immunologic causes of miscarriage and related histopathological features.

## 3. Materials and Methods

**Figure 1:**
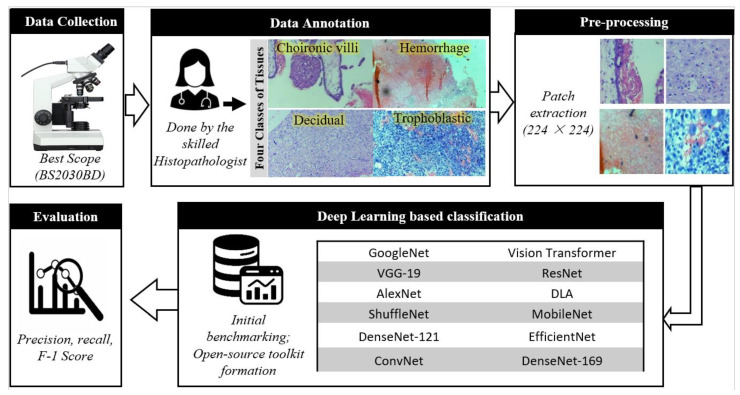
Illustration of the generic framework employed in this study.

The proposed framework for the classification of histopathology images began with data collection, where 5666 histopathological images were acquired under an IRB-approved protocol at Atia Hospital, Karachi, Pakistan. These images, sized 1280 × 729 pixels, contain representative tissue samples from clinical conditions. Following data collection, expert pathologists annotated the dataset to ensure reliable and accurate labels. Cases with discrepancies among pathologists were excluded to maintain consistency and minimize bias in the dataset. To standardize the data, preprocessing was performed by dividing the images into patches of size 224 × 224 pixels, ensuring uniform input dimensions for deep learning models. Patches with less than 50% tissue coverage were filtered out to enhance the dataset’s quality and relevance for training. Deep learning models were trained on the processed dataset, employing a 70/30 patient-level training-to-testing split to avoid data leakage. Model evaluation was carried out using precision, recall, and F1-score, offering a comprehensive assessment of classification accuracy and generalizability. This framework ensured systematic data handling and robust model development for histopathological image classification. Figure 1 presents the generic framework employed in this study, and the following subsection provides detailed descriptions of each step.

### 3.1. Dataset Acquisition and Annotation

The dataset was collected at Atia Hospital, Karachi, Pakistan under the AGH/IRB/2024/01. Histopathological samples were taken by the curetting sent by clinicians labeled as POC. A consultant histopathologist prepared digital images using a camera-connected microscope at 10× magnification, focusing on four types of pathological features commonly seen in routine clinical practice: chorionic villi, trophoblastic tissues, hemorrhage, and decidual tissue. Two additional histopathologists cross-checked the cases, and those with discrepancies between the pathologists were excluded from the dataset. This expert-driven classification provides a solid foundation for the model, ensuring that the ground truth labels are accurate and reliable. The dataset contains four types of tissues: chorionic villi, decidual tissue, hemorrhage, and trophoblastic tissue. The total number of cases for each tissue type is 204 (chorionic villi), 109 (decidual tissue), 136 (hemorrhage), and 101 (trophoblastic tissue). The original cases, sized 1280 × 729 pixels, were first divided into training and testing sets at the patient level, using a 70/30 training-to-testing ratio. This split ensured that images from the same patient did not appear in both the training and testing sets, preventing data leakage and ensuring the model’s ability to generalize to unseen data.

**Figure 2:**
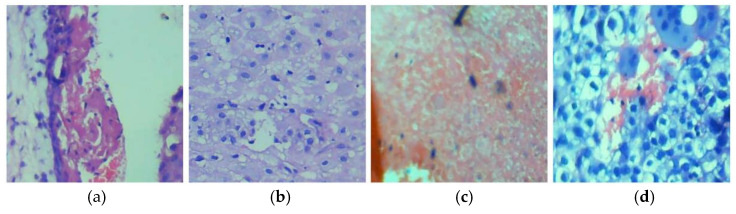
Example images from the dataset captured under 10× magnification using a camera-connected microscope. (**a**) Chorionic villi, (**b**) decidual tissue, (**c**) hemorrhage, and (**d**) trophoblastic tissue.

To standardize the dataset and facilitate model training, patches of size 224 × 224 pixels were extracted from the original images. This patch extraction process was designed to ensure consistent input size for the deep learning models while maintaining the relevant tissue information. During extraction, patches where the tissue section covered less than 50% of the patch area were filtered out to avoid misleading the model with empty or irrelevant regions. After patch extraction and filtering, the training set contained a total of 4155 samples (1391 chorionic villi, 926 decidual, 1138 hemorrhage, and 700 trophoblastic tissues), while the testing set included 1510 samples (390 chorionic villi, 349 decidual, 421 hemorrhage, and 350 trophoblastic tissues). The pathologists cross-checked the dataset to confirm the validity and accuracy of the selected patches, ensuring the dataset’s quality and consistency. In this study, the only preprocessing step performed was patch extraction, where images were divided into 224 × 224-pixel patches to standardize input sizes for the deep learning models. No additional preprocessing, such as color normalization or augmentation, was applied. This approach ensured the model learned directly from the natural variability in histopathological data. Figure 2 presents sample images from the dataset, while Table 1 summarizes key details about the proposed HistoPoC dataset.

### 3.2. Dataset Description

#### 3.2.1. Chorionic Villi

Chorionic villi [13] are finger-like projections that extend from the surface of the chorion, one of the membranes surrounding a developing embryo. These villi play a crucial role in the exchange of nutrients, gases, and waste products between the mother and the developing fetus during pregnancy. The villi are part of the placenta and contain blood vessels that help in the transfer of oxygen and nutrients from the mother’s blood to the fetal blood. In the context of histopathology, detecting and analyzing chorionic villi is important for diagnosing various conditions, such as placental abnormalities and pregnancy complications (e.g., preeclampsia), and for performing procedures like chorionic villus sampling (CVS), which is used for prenatal genetic diagnosis. Recent advancements in machine learning and computer vision have led to the development of automated techniques for detecting chorionic villi in histopathology images.

**Figure 3:**
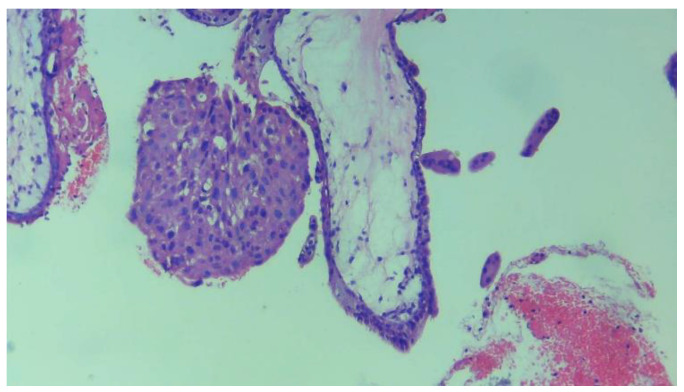
Example of chorionic villi tissue from the dataset captured under 10× magnification using a camera-connected microscope.

The advantages of automated detection using AI and deep learning models for detecting chorionic villi in histopathology images are substantial, particularly in the context of medical image analysis. Human pathologists may introduce variability in their assessments due to fatigue, subjective interpretation, or subtle image features. Automated detection systems provide more consistent and objective results, eliminating inter-observer variability. AI-based detection can be used to assist pathologists by highlighting suspicious areas (e.g., abnormal chorionic villi) in the images for further manual review. This can serve as a second opinion, helping improve diagnostic confidence. Automated systems can provide precise quantitative measurements of chorionic villi properties, such as size, shape, and distribution, which can be difficult and time-consuming to measure manually. Figure 3 presents a chorionic villi sample image from our curated dataset.

#### 3.2.2. Decidual Tissues

**Figure 4:**
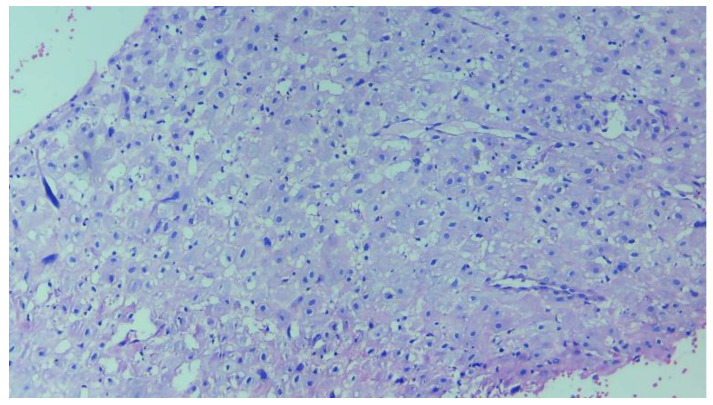
Example of decidual tissue from the dataset captured under 10× magnification using a camera-connected microscope.

Decidual tissue [14] refers to the specialized endometrial tissue that forms during pregnancy in the uterus. It plays a critical role in supporting the developing embryo and fetus, facilitating maternal–fetal exchange, and regulating the immune response to prevent rejection of the fetus. The decidual tissue is rich in glands, blood vessels, and immune cells and is involved in maintaining the pregnancy by providing structural and nutritional support. AI-powered detection of decidual tissues from histopathology images offers several advantages that enhance diagnosis, research, and clinical practice. AI models such as deep learning-based systems (e.g., CNNs or U-Net) can detect subtle abnormalities in decidual tissue that may be indicative of complications like placental insufficiency, preeclampsia, or miscarriage risks. AI can consistently identify patterns or abnormalities that may be challenging for human pathologists to detect, particularly in the early stages of pathology. AI systems provide consistent results in detecting and analyzing decidual tissue across different histopathology samples, reducing the variability and subjectivity that may arise from human interpretation. Figure 4 shows a sample decidual tissue from our dataset.

#### 3.2.3. Hemorrhage Tissues

**Figure 5:**
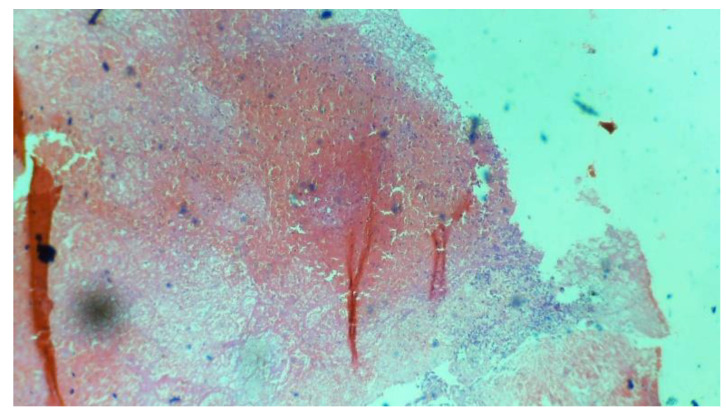
Example of hemorrhage tissue from the dataset captured under 10× magnification using a camera-connected microscope.

Hemorrhage [15] refers to bleeding or the escape of blood from a ruptured blood vessel into surrounding tissues. Hemorrhagic tissues are areas where bleeding has occurred, leading to the accumulation of blood in various tissues, which can be visible under a microscope in histopathology images. Hemorrhages can occur in different parts of the body and may result from trauma, vascular diseases, or medical conditions such as hypertension or blood clotting disorders. In histopathology, hemorrhagic tissues are generally identified by the presence of red blood cells (RBCs) outside the blood vessels, staining red or pink in Hematoxylin and Eosin (H&E)-stained slides. Pathologists manually identify hemorrhage by assessing the distribution and characteristics of the RBCs within the tissue. Automated detection of hemorrhage in histopathology images using AI and deep learning technologies offers several important advantages: AI models, especially those using deep learning, can detect subtle signs of hemorrhage that may not be easily visible to human pathologists, especially in early or microscopic forms (e.g., petechiae). This can help in the early diagnosis of conditions associated with vascular fragility or coagulopathies. Manual identification of hemorrhage can be subjective, leading to variability between pathologists. AI models can consistently and objectively detect hemorrhagic areas in tissue samples, reducing human error and increasing diagnostic confidence. Early, small-scale hemorrhages (e.g., microbleeds in the brain or in placental tissues) may be difficult to detect manually. AI models can be trained to spot these early signs, allowing for timely intervention in cases such as preeclampsia, cerebral small vessel disease, or early-stage cancer. Figure 5 presents a sample of hemorrhage tissue from the dataset.

#### 3.2.4. Trophoblastic Tissues

**Figure 6:**
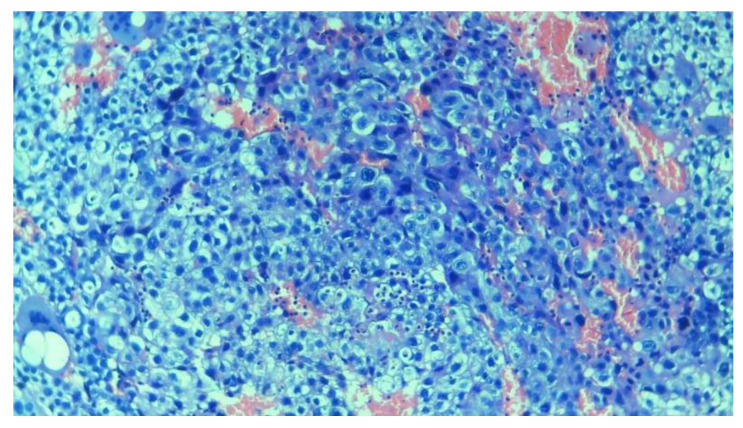
Example of trophoblastic tissue from the dataset captured under 10× magnification using a camera-connected microscope.

Trophoblastic tissues [16] refer to the cells that form part of the outer layer of the blastocyst in early embryonic development. These tissues play a crucial role in the formation of the placenta and are responsible for implantation in the uterine wall, nutrient exchange, and secretion of hormones necessary for maintaining pregnancy. Trophoblastic cells can differentiate into various cell types, primarily contributing to the development of the chorion and the placenta, essential for fetal–maternal interaction. Automated detection of trophoblastic tissues through AI and deep learning models offers several advantages in clinical diagnosis and research, especially in pregnancy-related conditions such as trophoblastic diseases, placental abnormalities, and implantation issues. Deep learning models can provide consistent and objective assessments of trophoblastic tissues, reducing human diagnostic errors. AI can quickly analyze large datasets, highlighting abnormal growth patterns that may indicate trophoblastic tumors or molar pregnancies. Trophoblastic cells are key to placental development. AI models can assist in identifying abnormalities in the trophoblast layer, such as insufficient trophoblastic invasion, which can lead to pregnancy complications like preeclampsia, placental insufficiency, or intrauterine growth restriction (IUGR). AI can quantify the invasion of extravillous trophoblasts into the maternal decidua and blood vessels, helping to assess whether proper placentation has occurred. Inadequate invasion can lead to poor placental perfusion and subsequent fetal growth problems. Figure 6 shows a sample of trophoblastic tissue from the dataset.

### 3.3. Baseline Models

In this study, we employed a variety of baseline models to establish a comprehensive framework for evaluating the performance of our proposed AI techniques in histopathological examination. These baseline models served as critical references, enabling us to assess the advancements brought about by our approach in detecting and classifying tissue phenotypes in POC after spontaneous abortion. The following subsections detail the specific baseline models utilized in our research.

#### 3.3.1. GoogleNet

GoogleNet [17] is a deep convolutional neural network architecture that introduces the inception module for image classification. This design allows the network to effectively learn multi-scale features by combining convolutions of different kernel sizes within a single layer. GoogleNet stacks several inception layers, improving the efficiency of parameter usage while maintaining high classification accuracy. Key features include the following:

- Inception Modules: combine multiple convolution layers with different kernel sizes;
- Global Average Pooling: reduces the spatial dimensions of the output, aiding in efficient parameter usage;
- Auxiliary Classifiers: used to prevent overfitting by providing additional gradients during backpropagation.

#### 3.3.2. VGGNet

VGGNet [18] is a well-known deep CNN architecture recognized for its simplicity and depth. It primarily consists of a series of convolutional layers (with 3 × 3 filters) followed by max-pooling layers. The architecture is uniform, with a clear focus on depth, making it computationally intensive but highly effective for large-scale datasets like ImageNet. VGGNet provides a solid baseline due to the following:

- Deep Architecture: stacks many layers to learn hierarchical features;
- Small 3 × 3 Filters: efficient for capturing fine-grained features in images;
- Max-Pooling Layers: used to reduce spatial dimensions and control overfitting.

#### 3.3.3. AlexNet

AlexNet [19] is a pioneering CNN architecture that revolutionized deep learning with its success in the ImageNet competition. AlexNet introduces several key innovations:

- Group Convolution: divides input and kernel channels into separate groups, significantly reducing the number of parameters;
- Multiple Convolutional Layers: help capture spatial features across different regions of the image;
- GPU Utilization: AlexNet was one of the first models to leverage GPUs for training deep neural networks, which enabled the training of deeper models on large datasets.

#### 3.3.4. ShuffleNet

ShuffleNet [20] is designed to be highly efficient, specifically targeting mobile devices. It addresses the computational inefficiencies of 1 × 1 convolutions by using the following:

- Group Convolutions: split convolutions into smaller groups, reducing computation;
- Channel Shuffle Operation: enhances information flow between feature channels;
- Depthwise Convolutions: applied to the 3 × 3 convolution layers to reduce computational costs;
- ShuffleNet is suitable for devices with limited computational power while maintaining reasonable accuracy.

#### 3.3.5. DenseNet

DenseNet [21] improves the flow of information and gradients across layers by connecting each layer to every other layer in a dense block. DenseNet-169 functions as follows:

- Dense Blocks: every layer receives input from all preceding layers;
- Fewer Parameters: each layer only needs to learn new features, as it can use features from previous layers;
- Better Gradient Flow: makes it easier to train deeper networks;
- DenseNet-169 optimizes both depth and efficiency while reducing the number of parameters compared to traditional CNNs.

#### 3.3.6. ConvNet

ConvNet [22] is a more general architecture that integrates depthwise separable convolutions and lightweight operations for enhanced computational efficiency. Its design focuses on the following:

- Depthwise Separable Convolutions: reduce the number of parameters and computation;
- Interpretability and Robustness: built to ensure that the network can provide interpretable results, making it suitable for medical applications;
- Efficient Feature Extraction: balances computation and feature.

#### 3.3.7. Vision Transformer

Vision Transformer (ViT) [23] adapts the transformer model, initially developed for natural language processing (NLP), for image recognition tasks. ViT functions as follows:

- Image Patches: the image is split into fixed-size patches, which are then linearly embedded;
- Transformer Architecture: captures long-range dependencies in images;
- Scaling: Demonstrates superior performance when trained on large datasets, making it effective for complex image recognition tasks.

ViT outperforms traditional CNNs in certain image recognition benchmarks.

#### 3.3.8. ResNet

Residual Neural Network (ResNet) [24] introduces the concept of residual learning to address the degradation problem in very deep networks. ResNet uses the following:

- Residual Connections: skip connections allow gradients to flow more effectively during backpropagation;
- Deep Architecture: enables networks with up to 152 layers to be trained effectively;
- Identity Mapping: helps preserve information across layers.

ResNet has become one of the most widely used architectures due to its effectiveness in training very deep networks.

#### 3.3.9. DLA

Deep Layer Aggregation (DLA) [25] improves performance by aggregating features across layers:

- Iterative Deep Aggregation (IDA): combines features starting from the shallowest layers and progressively merges deeper features;
- Hierarchical Deep Aggregation (HDA): merges blocks and stages in a hierarchical manner, enhancing feature learning;
- Improved Accuracy: allows the network to learn richer feature combinations with fewer parameters;
- DLA is efficient in both accuracy and computational cost.

#### 3.3.10. MobileNet

MobileNet [26] focuses on creating lightweight models that are ideal for mobile and embedded vision applications. Key features include the following:

- Depthwise Separable Convolutions: reduces computational cost;
- Trade-off Between Latency and Accuracy: two global hyperparameters allow for model size customization based on application constraints;
- Versatility: suitable for applications such as object detection, fine-grained classification, and geo-localization;
- MobileNet is well suited for mobile and embedded systems that require efficient processing.

#### 3.3.11. EfficientNet

EfficientNet [27] is a highly efficient CNN that uses a compound scaling method to uniformly adjust the network’s width, depth, and resolution. Its key characteristics include the following:

- Compound Scaling: balances width, depth, and resolution to optimize performance;
- Better Accuracy: outperforms previous models on benchmarks like ImageNet;
- Efficiency: designed for resource-constrained settings, making it suitable for mobile and embedded applications;
- EfficientNet provides a better balance of performance and computational efficiency compared to previous architectures.

These baseline models form the foundation of our comparative analysis and allow us to benchmark the performance of our proposed AI techniques in the context of histopathological image analysis.

## 4. Results

### 4.1. Training

The training of the state-of-the-art models was conducted using the PyTorch framework [28], which provides flexibility and dynamic computation graphs, making it suitable for deep learning research. The hardware setup included a Core-i7-7700 CPU running at 3.60 GHz, 24 GB of RAM, and an NVIDIA GeForce GTX 1070 GPU [29] with 8 GB of random access memory. This configuration offered the necessary computational power for handling deep learning tasks. The models were initialized with pretrained weights instead of being trained from scratch. By utilizing pretrained models, the training process benefitted significantly from leveraging prior knowledge. PyTorch was chosen for its ease of use and the flexibility it provides in building custom models and experimenting with different architectures. The training process used the Adam optimizer [30], which adjusts learning rates for each parameter dynamically, helping to balance the trade-off between convergence speed and stability. A learning rate of 0.0001 was set, which is a common choice, offering a smooth learning curve without being too aggressive or too slow. The models were trained with a batch size of 16, which allowed efficient use of the available GPU memory while maintaining stability in the gradient updates. The training was conducted over 100 epochs to ensure the models had ample time to converge and fully capture the nuances of the dataset. To prevent overfitting, an early stopping mechanism was implemented. Early stopping is particularly effective in situations where the model begins to overfit the training data after a certain number of epochs.

### 4.2. Key Metrics for Performance Analysis

Evaluation metrics help assess how well a model performs across multiple classes, providing insight into its strengths and weaknesses for each class. In this study, we used precision, recall, and F1-score to evaluate the performance of the state-of-the-art models on our dataset. Precision measures the accuracy of positive predictions, indicating the proportion of correctly predicted instances for a class compared to all instances predicted as that class. It helps to assess how well the model avoids false positives. Recall measures the ability of the model to correctly identify all actual positive instances, providing insight into how well the model avoids missing positive cases (false negatives). F1-score is the harmonic mean of precision and recall, offering a balanced measure that accounts for both false positives and false negatives. It is particularly useful when the class distribution is imbalanced, as it reflects the trade-off between precision and recall. Equation (1)–(3) represents precision, recall, and F1-score, respectively.

(1) $$\mathrm{Precision}=\frac{1}{K}\sum_{j=1}^{K}\frac{{TP}_{j}}{{TP}_{j}+{FP}_{j}}$$

(2) $$\mathrm{Recall}=\frac{1}{K}\sum_{j=1}^{K}\frac{{TP}_{j}}{{TP}_{j}+{TN}_{j}},$$

(3) $$F1-\mathrm{score}=2\times \frac{Precision\times Recall}{Precision+Recall}$$

In Equations (1)–(3), *K* denotes the number of classes in the dataset; ${TP}_{j}$ indicates the total number of correct predictions; ${FP}_{j}$ denotes the total number of false positives; ${TN}_{j}$ represents the total number of true negatives; and ${FN}_{j}$ indicates the total number of false negatives.

### 4.3. Results of State-of-the-Art Models

In this section, we evaluate and compare the performance of several state-of-the-art deep learning models based on their precision, recall, and F1-score, as shown in Table 2. Among the models tested, MobileNet [26] emerged as the top performer, achieving the highest precision (0.7864), recall (0.7856), and F1-score (0.7788). This outstanding performance can be attributed to its efficient architecture, which strikes an optimal balance between accuracy and computational cost, making it particularly well suited for resource-constrained environments such as mobile and embedded systems. A key feature of MobileNet is its use of depthwise separable convolutions, a technique that significantly reduces the number of parameters and the computational complexity of the network. Unlike traditional convolutional neural networks (CNNs) such as VGG-19 and ResNet-50, which rely on standard convolutions, MobileNet’s factorized convolutions allow for more efficient extraction of features from images while maintaining high levels of accuracy. This efficiency leads to enhanced precision, as MobileNet can focus on relevant features using fewer computational resources, ultimately resulting in more accurate predictions.

In summary, while earlier models like GoogleNet and AlexNet still offer reasonable performance, more recent models, including ResNet, EfficientNet, and especially MobileNet, have demonstrated significant advancements in both accuracy and efficiency. MobileNet stands out as the leading model in terms of precision and recall, making it an ideal choice for tasks that demand both high performance and low computational overhead. Its architectural innovations and ability to maintain accuracy in constrained environments ensure that it remains at the forefront of modern deep-learning techniques.

## 5. Discussion

The results of this study demonstrate the significant potential of artificial intelligence (AI) in enhancing the histological diagnosis of products of conception. AI techniques can assist histopathologists in accurately identifying tissue phenotypes, particularly chorionic villi, which are crucial for diagnosing miscarriages or abortions. The application of AI in histopathological examinations has been shown to improve diagnostic accuracy by reducing subjectivity and inter-observer variability. The use of AI-driven models can provide consistent assessments, thereby enhancing the reliability of the diagnostic process. Given the frequency with which tissue specimens from miscarriages and abortions are received by pathology laboratories, the integration of AI can streamline the workflow, allowing for more efficient processing and analysis of these specimens. This is especially important in high-throughput environments. The study highlights the capacity of AI systems to learn and improve automatically from experience and available data. This capability enables continuous enhancement of the diagnostic process, making AI an invaluable tool for pathologists in the long term. AI can serve as an effective decision-support system for histopathologists, assisting them in the microscopic assessment of slides and improving their overall diagnostic capabilities. This collaborative approach between human expertise and AI can lead to better patient outcomes. The findings underscore AI’s role in the ongoing digital revolution within medical imaging and diagnostics. By integrating AI into histopathology, the study contributes to a broader movement toward more advanced, automated, and data-driven medical practices. This research lays the groundwork for future studies exploring the full potential of AI in histopathology. The dataset and models developed can serve as a basis for further investigations into other tissue phenotypes and applications in different medical contexts. The insights gained from this study have the potential to inform clinical decision making, enhance the understanding of spontaneous abortion causes, and guide the development of novel treatments and interventions. The key findings of this study are as follows:

- The results of this study demonstrate the significant potential of artificial intelligence (AI) in assisting the histological diagnosis of products of conception. AI techniques can assist histopathologists in accurately identifying tissue phenotypes, particularly chorionic villi, which are crucial for diagnosing miscarriages or abortions;
- The application of AI in histopathological examinations has been shown to improve diagnostic accuracy by reducing subjectivity and inter-observer variability. The use of AI-driven models can provide consistent assessments, thereby enhancing the reliability of the diagnostic process;
- Given the frequency with which tissue specimens from miscarriages and abortions are received by pathology laboratories, the integration of AI can streamline the workflow, allowing for more efficient processing and analysis of these specimens. This is especially important in high-throughput environments;
- The study highlights the capacity of AI systems to learn and improve automatically from experience and available data. This capability enables continuous enhancement of the diagnostic process, making AI an invaluable tool for pathologists in the long term;
- AI can serve as an effective decision-support system for histopathologists, assisting them in the microscopic assessment of slides and improving their overall diagnostic capabilities. This collaborative approach between human expertise and AI can lead to better patient outcomes;
- The findings underscore AI’s role in the ongoing digital revolution within medical imaging and diagnostics. By integrating AI into histopathology, the study contributes to a broader movement toward more advanced, automated, and data-driven medical practices;
- This research lays the groundwork for future studies exploring the full potential of AI in histopathology. The dataset and models developed can serve as a basis for further investigations into other tissue phenotypes and applications in different medical contexts;
- The insights gained from this study have the potential to help clinical decision-making, enhance the understanding of spontaneous abortion causes, and guide the development of novel treatments and interventions.
- Our study also has some potential weaknesses, which can be listed as follows:
- The study primarily focuses on internal validation of the AI models. External validation using independent datasets from different institutions would provide a more robust assessment of the models’ generalizability and real-world applicability;
- While the dataset comprises 5666 annotated images, it is sourced from a single institution. This limited geographical and demographic diversity may affect the generalizability of the findings to other populations or clinical settings;
- The accuracy of the AI models is heavily reliant on the quality of annotations provided by the three expert pathologists. Any potential biases or discrepancies in their classification could impact the training and performance of the models, leading to inconsistencies in diagnostic accuracy;
- Depending on the distribution of different tissue phenotypes in the dataset, there may be an imbalance in the number of images representing each phenotype. This imbalance can affect the model’s ability to learn effectively from underrepresented classes, potentially leading to suboptimal performance in identifying those tissue types;
- The study primarily focuses on identifying chorionic villi and other specific tissue phenotypes, which may limit the broader applicability of the findings to other histological diagnoses or conditions.

## 6. Conclusions

This study highlights the transformative potential of artificial intelligence in enhancing the histopathological examination of POC following spontaneous abortion. By leveraging advanced AI techniques, we demonstrated that tissue phenotyping can achieve higher accuracy and reliability compared to traditional manual methods. The development of our novel dataset, comprising 5666 annotated images, not only provides a valuable resource for further research but also serves as a benchmark for evaluating AI models in this critical area of medical diagnostics. Our findings indicate that AI-driven approaches show promising potential for improving diagnostic accuracy. However, it is important to note that these conclusions were based on internal validation, and further external validation using independent datasets from different institutions is necessary to fully assess the generalizability and real-world applicability of the models. Nonetheless, our research suggests that AI can enhance the diagnostic process, contributing valuable insights into the causes of spontaneous abortion. Future studies will help confirm the broader applicability of these models and their integration into routine histopathological practice. The successful validation of deep learning models within this study underscores the viability of integrating AI into routine histopathological practice, marking a significant step forward in the field of tissue phenotyping. As we continue to explore the intersection of AI and medical diagnostics, our research contributes to a growing body of evidence supporting the adoption of intelligent systems in healthcare, paving the way for future advancements in the understanding and management of spontaneous abortion.

## Figures and Tables

**Table 1:** Key details of the HistoPoC dataset.

Attribute	Details
Dataset Name	HistoPoC
Source	Atia Hospital, Karachi, Pakistan
IRB Approval	AGH/IRB/2024/01
Sample Collection Method	Histopathological samples obtained by curetting sent by clinicians, labeled as POC
Magnification	10× magnification using a camera-connected microscope
Pathological Features	Chorionic villi, trophoblastic tissue, hemorrhage, and decidual tissue
Data Exclusion Criteria	Cases with discrepancies among pathologists were excluded from the dataset
Total Number of Cases	550 (204 chorionic villi, 109 decidual tissue, 136 hemorrhage, and 101 trophoblastic tissue)
Image Dimensions	1280 × 729 pixels
Dataset Split	70/30 training-to-testing ratio at the patient level, ensuring no data leakage
Patch Size	224 × 224 pixels
Patch Extraction Process	Patches were extracted to standardize input size, filtering out patches with less than 50% tissue coverage
Post-Extraction Verification	Pathologists cross-checked extracted patches for validity and accuracy before inclusion in the dataset
Training Set	1391 chorionic villi, 926 decidual, 1138 hemorrhage, and 700 trophoblastic tissues
Testing Set	390 chorionic villi, 349 decidual, 421 hemorrhage, and 350 trophoblastic tissues

**Table 2:** Performance comparison among baseline models.

Methods	Precision	Recall	F1-Score
GoogleNet [17]	0.6046	0.6612	0.6075
VGG-19 [18]	0.7585	0.7313	0.7060
AlexNet [19]	0.7194	0.7313	0.7147
ShuffleNet [20]	0.7322	0.7201	0.7167
DenseNet-121 [21]	0.7304	0.7346	0.7237
ConvNet [22]	0.7364	0.7439	0.7314
Vision Transformer [23]	0.7418	0.7439	0.7393
EfficientNet [27]	0.7612	0.7684	0.7570
DenseNet-169 [21]	0.7622	0.7624	0.7575
ResNet-50 [24]	0.7641	0.7704	0.7623
DLA [25]	0.7795	0.7829	0.7771
MobileNet [26]	**0.7864**	**0.7856**	**0.7788**

## Data Availability

This paper discloses a public image dataset that can be accessed by sending an email to the corresponding author.
